# The Detection of Fosfomycin-Modifying Enzymes (fos) in Uropathogenic Enterobacterale, Azerbaijan, Iran

**DOI:** 10.1155/2023/3766269

**Published:** 2023-05-19

**Authors:** Aidin Lalezadeh, Pardis Ghotaslou, Reza Ghotaslou

**Affiliations:** ^1^Department of Microbiology, Faculty of Medicine, Tabriz University of Medical Sciences, Tabriz, Iran; ^2^Students' Research Committee, Tabriz University of Medical Sciences, Tabriz, Iran; ^3^Immunology Research Center, Tabriz University of Medical Sciences, Tabriz, Iran

## Abstract

*Enterobacteriaceae* is the most common agent of urinary tract infections (UTIs). Multidrug resistant (MDR) and XDR (extensively drug-resistant) Enterobacteriaceae in UTIs have increased in the world. The present study aimed to study the fosfomycin resistance frequency and the fosfomycin resistance genes among Enterobacteriaceae isolated from UTIs. The urine was collected and cultured in the standard protocol. To determine the susceptibility testing to fosfomycin in 211 isolates, agar dilution and disk agar diffusion methods were used. MDR was nonsusceptibility to at least one agent in three or more antimicrobial categories. The fosfomycin resistance genes were also evaluated by PCR. The frequency of resistance to fosfomycin was in 14 (6.6%) and 15 (7.1%) isolates by the disk agar diffusion and MIC assays, respectively. However, the MIC_50_ and MIC_90_ existed at 8 *μ*g/mL and 16 *μ*g/mL, respectively. The MDR was found in 80%. The frequencies of fosfomycin resistance genes were 5 (33.3%), 3 (20%), 2 (13.3%), 1 (6.6%), and 1 (6.6%) for fosC, fosX, fosA3, fosA, and fosB2, respectively. The fosB and fosC2 were not found. A low resistance rate to fosfomycin is observed. Fosfomycin is still one of the most effective and valuable alternative antibiotics against MDR *Enterobacteriaceae* isolated from UTIs in our region.

## 1. Introduction

The most common cause of urinary tract infections (UTIs) is *Enterobacteriaceae*. Recently, the occurrence of resistance to antimicrobial agents among *Enterobacteriaceae* and multidrug resistant (MDR) and extensively drug-resistant (XDR) isolates have become a worldwide concern. The increased resistance rate to antibiotics has directed to a decrease in the successful management of UTIs due to Enterobacteriaceae [1]. A few of the causes of the increase in MDR or XDR pathogens are empirical antibiotic prescription without urine culture and susceptibility testing and ineffective UTI treatment as well as the development of persistent or recurrent infections [2]. Enterobacteriaceae as heterogeneous groups cause numerous infections mainly UTIs, bacteremia, hospital infections, and gastroenteritis [3, 4]. UTIs are considered one of the most common bacterial infections seen in primary health care and are very common especially in females [5]. In the United States, UTIs are considered a huge economic problem that costs about $6 billion per year [6].

During the past decade, the introduction of novel antimicrobial agents has been restricted and physicians sometimes have to use some old antimicrobial agents that have come out of the clinical practice. Nevertheless, fosfomycin rests on an actual antimicrobial agent against the UTIs caused by *Enterobacteriaceae* [7, 8]. Fosfomycin is a bactericidal antibiotic that inhibits the production of bacterial peptidoglycan (inhibits UDP-N-acetylglucosamineenolpyruvyl transferase) [9]. Fosfomycin has an exceptional mechanism [10] and is effective on Gram-negative and Gram-positive bacteria [11]. The use of fosfomycin is approved in uncomplicated UTIs by the FDA [12]. There are three forms of fosfomycin including tromethamine as a soluble salt, calcium for oral use in UTI treatment, and disodium for intravenous usage [13]. A previous study stated high susceptibility to fosfomycin in MDR and XDR Enterobacteriaceae [1]; on the other hand, Oteo et. al reported resistance to fosfomycin among Enterobacteriaceae isolates [14]. Resistance to fosfomycin has often been prompted by plasmid genes, including fosfactors [15]. In the present study, the resistance frequency of Enterobacteriaceaeto fosfomycin and the fosfomycin resistance genes were studied.

## 2. Materials and Methods

### 2.1. Bacterial Isolates

Two hundred and eleven Enterobacteriaceae isolated from UTIs were collected from hospitalized patients from June to December 2021 in Azerbaijan, Iran. The samples were collected from patients who had signs and symptoms suggestive of UTIs as decided by the physician. Patients were recommended to collect midstream urine samples into a sterile wide-mouth container with all aseptic measures. Samples from catheterized patients were occupied with aseptic measures. The collected urine was tested within an hour of sampling. The urine samples were cultured by a semiquantitative method on blood agar and MacConkey agar (Merck, Germany) and incubated at 37 C for 24 hours. Only those samples which grew significant bacteriuria were included in the study. A sample was considered positive for UTIs if a single organism was cultured at a concentration of 10^5^ cfu/mL. Finally, bacterial identification was performed based on Gram staining, culture, and standard biochemical tests such as TSI, H2S, citrate, indole, urea, lysine, SIM, MR-VP, and gas production [16, 17]. All fungal pathogens other than Enterobacteriaceae isolates were excluded. The patients have taken antibiotics within the last two weeks before this study, the patients who did not give their consent for participation, women who were in their menstrual period, and those people who did not have the conditions to enter the study were excluded. The current study was approved by the Ethics Committee of Tabriz University of Medical Sciences (Ir.Tbzmed.Rec.1397.579).

### 2.2. The Susceptibility Testing

The susceptibility testing was done by the disk agar diffusion method concerning the Clinical and Laboratory Standards Institute (CLSI) guidelines [18]. The antibiotic disks were trimethoprim/sulfamethoxazole, nitrofurantoin, fosfomycin, amoxicillin-clavulanic acid, imipenem, cefixime, ampicillin, cefazolin, amikacin, gentamicin, nalidixic acid, ciprofloxacin, and levofloxacin (Mast, UK). The fosfomycin disk (200 *μ*g) enclosed 50 *μ*g (glucose-6-phosphate). The MICs (minimum inhibitory concentrations) of fosfomycin were carried out via the agar dilution assay supplemented with 25 *μ*g/mL of glucose-6-phosphate. The analysis of MIC results was prepared as stated by the CLSI guiding principles [18]. The agar dilution method (Mueller–Hinton Broth) was used to measure MIC under defined test conditions; MIC is defined as the lowermost concentration of an antibacterial agent that inhibits the growth of bacteria. The method described in this research was planned for the testing of pure cultures of bacteria that are easily grown by overnight incubation. The antimicrobial powders (Merck, Germany) were prepared by the manufacturer. The MIC of fosfomycin was done by supplementation with 25 mg/L glucose-6-phosphate. To control the susceptibility testing, we used *E. coli* ATCC 25922. Multiple drug resistance (MDR) is resistance to at least one antibiotic in three or more antimicrobial classes (1). XDR was defined as no susceptibility to at least one agent in wholly but two or fewer antimicrobial groups (i.e., bacterial isolates persist susceptible to only one or two antimicrobial categories) (8).

### 2.3. The PCR

The DNA extraction of isolates was carried out by the boiling method [19]. To extract the DNA, samples were incubated at 99°C for 15 min, with adding 0.5% Tween 20, and immediately cooled on ice. The fos genes were detected by PCR. PCR assays were achieved on a 96-well Thermal Cycler (Eppendorf, UK). PCR was carried out in the reactions with a last volume of 20 *μ*L including 5 *μ*L of Master Mix 1X (SinaClon Co., Iran) having Taq DNA polymerase, MgCl_2,_ and dNTPs. Primers and conditions were used as previously described by Bi et al. [20]. Resistance to fosfomycin has been frequently encouraged by plasmid genes, including fos factors [14]. PCR products were examined via electrophoresis on a 1% agarose gel [21].The gels were marked with the DNA-safe stain and were imagined underneath ultraviolet light; the size of the products was measured by judgment with a 100 bp molecular size marker (SinaClon Co., Iran). In the current study, 22 randomly designated fosfomycin-sensitive isolates were used as controls. Furthermore, each of the positive-fosfomycin resistance gene strains in our laboratory was used as a positive control. One negative control was also involved in each PCR run, where DNA was substituted by an equal volume of sterilized water.

### 2.4. Statistical Analysis

The data was analyzed using descriptive-analytic assays in SPSS software version 22 (Washington, the USA). Qualitative data were assessed by using Chi-square and Fisher's test and *P* values ≤ 0.05 were considered statistically significant.

## 3. Results

In the present study, 211 nonduplicates, Enterobacteriaceae, were isolated from 67 males (31.8%) and 142 females (68.2%). The mean age of patients was 41 ± 20 years. The isolates were collected from the various hospital wards. In the present study, 158 (74.9%) of isolates were *Escherichia coli* (*E. coli*), followed by *Klebsiella pneumoniae* (*K. pneumonia*) 39 (18.5%), *Enterobacter*agglomerans 4 (1.9%), *Enterobacter cloacae* 2 (0.9%), *Klebsiella oxytoca*, 2 (0.9%), *Proteus vulgaris* 2 (0.9%), *Morganellamorganii* 2 (0.9%), and *Proteus mirabilis 2* (0.9*%*).

**Figure 1:**
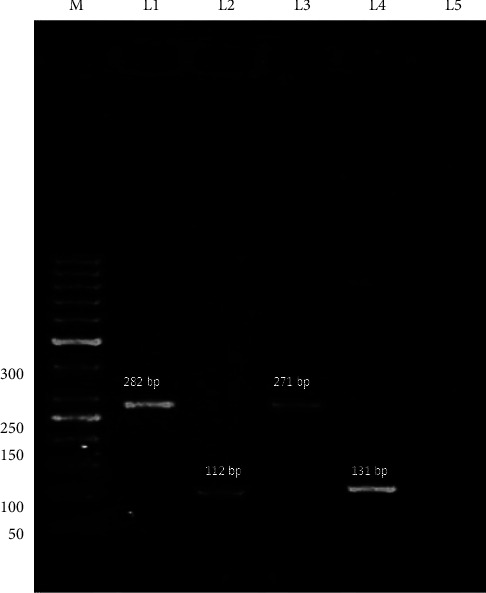
The gel electrophoresis of fosfomycin resistance genes in 1% agarose gel. Lane M DNA size marker (50 bp); L1: fosA3 gene (282 bp), L2: fosC gene (112 bp), L3: fosA gene (271 bp), L4: fosX gene (131 bp), and L5: negative.

The highest rate of resistance was detected to ampicillin (95.1%), followed by cotrimoxazole (77.3%), nitrofurantoin (75%), nalidixic acid (70%), amoxicillin-clavulanic acid (70%), cefixime (65%), cefazolin (60%), ciprofloxacin (50%), levofloxacin (44%), gentamicin (43.5%), amikacin (17.3%), imipenem (9.2%), and fosfomycin (6.6%) (Table 1). The MDR was found in 80%. The frequency of resistance to fosfomycin was 14 (6.6%) by the disk agar diffusion assay. The resistance rate to fosfomycin was found in 15 isolates (7.1%) by MIC (9 *E. coli* and 6 *K. pneumonia* isolates). The MIC range was 1–256 *μ*g/mL and the MIC_50_ and MIC_90_ were 8 *μ*g/mL and 16 *μ*g/mL, respectively (Table 2). The resistance to fosfomycin among MDR isolates was higher than non-MDR, 10% and 6%, respectively (Pv ≤ 0.05). The rate of fosfomycin resistance genes in fosfomycin-resistant isolates was 5 (33.3%), 3 (20%), 2 (13.3%), 1 (6.6%), and 1 (6.6%) for fosC, fosX, fosA3, fosA, and fosB2, respectively. The fosB and fosC2 were not identified (Table 3 and Figure 1).

## 4. Discussion

Enterobacteriaceae is the most common causative agent of UTIs, and the rate of antibiotic resistance and MDR has increased [22]. In the current study, the MDR was high among Enterobacteriaceae isolated from UTIs. Fosfomycin is one of the most effective and useful drugs in the treatment of infections due to MDR Enterobacteriaceae.

Fosfomycin has widely distributed in many human organs, chiefly the urinary tract [23]. In the current research, the overall resistance rate to fosfomycin among Enterobacteriaceae was 7.1% by MICs. Falagas et al. in Greece showed that 8.2% of MDR Enterobacteriaceae isolates were resistant to fosfomycin [1]. These similar findings are also reported in different countries and Iran [8, 10, 23–26]. In the present study, the most fosfomycin-resistant isolates belonged to *K. pneumoniae* (23%) and *E. coli* (6.9%).The difference in the rate of resistance to fosfomycin may be due to the amount of antibiotic usage in the different are as depending on a diverse policy. However, one of the reasons for the increase in drug resistance is an inadequate empirical antibiotic prescription without urine culture and susceptibility testing. The use of prophylactic antimicrobial therapy is also considered the main risk factor in the development of antibiotic resistance.

In the current study, the highest resistance rate is observed to ampicillin (95.1) followed by trimethoprim/sulfamethoxazole (77.3). A high resistance rate to antimicrobial agents has already been described [27–29]. The great usage of antimicrobial agents in experimental practice leads to resistance of bacteria to antibiotics. Bacteria usually have two resistance mechanisms to antibiotics named innate and acquired. Acquired resistance gets up from chromosomal mutations, but resistance to antimicrobial agents is usually related to gene transfer (plasmids, transposons, and integrons or a combination of these mechanisms). Efflux pumps are also accepted as the chief cause of the MDR mechanism in bacteria. Enterobacteriaceae species are opportunistic pathogens in hospitals, and due to Enterobacteriaceae resistance to antibiotics, they are described as one of the leading reasons for resistant nosocomial infection [28].

The fosA3 gene was distinguished in 9% of isolates in China, whereas fosC2 and fosA genes were not found [30]. The fosA3 gene was also reported as the most common resistance gene among *E. coli* isolates in Hong Kong and China [30, 31]. Villa et al. found that fosA3 is a plasmid-mediated gene based on sequencing data, which is common in Asia and rare in European countries [32]. Zaniani et al. from Iran reported that the fosC2 and fosA3 genes were not detected in *E. coli* isolates [33]. However, there are several reports of fosfomycin resistance genes in Iran [28, 34–37]. In the current study, the rate of the fosA3 (0.9%) is not consistent with mentioned studies. The main fosfomycin resistance genes in the current study were fosC and fosX. The results of this study identified fosX gene as an important issue because this gene has been reported rarely in previous investigations.

The mobility of these genes (encoded on a conjugated plasmid, transposons, or within integrons) may accelerate the dissemination of fosfomycin resistance around our region. Mobile fosfomycin-resistant genes have also been distinguished in human, animal, food, and environmental source which initiate a growing worry regarding the risk of the spread of such bacteria, especially *E. coli*, at the human-animal-environment crossing point. The reason for the presence of fosC and fosX genes in the present study isolates as compared to their absence or low rate in previous studies may be due to the hospital setting, patient's population, geographic variation, sample size, the mechanism of antimicrobial resistance, kind of bacteria, and source of infections.

Today, it is suggested to use fosfomycin for the management of UTIs, particularly in severe UTIs in women and MDR Enterobacteriaceae [12, 38, 39]. Fosfomycin is an intensely effective drug against the uropathogenic MDR Enterobacteriaceae [40, 41].We must be aware that due to the wide spreading of resistant *Enterobacteriaceae* isolates to antibiotics in different places (animals, humans, food, and environment), as well as fosfomycin usage in veterinarians, *Enterobacteriaceae* is readily transferred to new places and other bacteria [30, 42]. Furthermore, considering the determinant locations of mobile genetic elements (for example, plasmids), even the minor recognized resistance cases are important. So, attributable to a high chance of resistance agents spreading, the prevention is chief strategy. One of the most main options for the prevention of resistance spreading to antibiotics may be combination therapy. The significant increase in MDR uropathogenic *Enterobacteriaceae* is considered a big challenge in the treatment of UTIs. On the other hand, high fosfomycin susceptibility among MDR isolates observed in the current study gives hope in using this drug, rather than using other nephrotoxic drugs such as colistin. Owing to the unique mechanism of fosfomycin action, low incidence of resistance, oral availability with single-dose administration, and less propensity to display cross-resistance to other antibiotics, fosfomycin is a good choice for the treatment of UTIs. It is still possible to prescribe fosfomycin in this area, but monitoring is necessary to understand the evolution of the dispersal of fos genes or other resistance mechanisms using advanced techniques such as Next Generation Sequencing.

To validate the results of the current study, it recommends doing a larger study targeting geographically and in diverse isolates. Our study also showed that disc diffusion agar is a good assay for fosfomycin susceptibility testing. Continuous monitoring of fosfomycin susceptibility is recommended to keep a check on any increase in resistance pattern, prevent its spread, and further aid in its clinical application. Despite our promising results, this study had some limitations such as the lack of examining resistance genes other than fos genes, the plasmid content, and the genetic background of the fos genes.

## 5. Conclusion

The MDR is worrying among *Enterobacteriaceae* isolated from UTIs, whereas the fosfomycin resistance rate is still low. The most common fosfomycin resistance genes belonging to *K. pneumoniae* and *E. coli* were fosC, fosX, fosA3, fosA, and fosB2.

## Figures and Tables

**Table 1:** The resistance rate of bacterial species to antimicrobial agents.

Bacteria	Fosfomycin (%)	Gentamicin (%)	Amikacin (%)	Ciprofloxacin (%)	Ampicillin (%)	Cotrimoxazol (%)	Nalidixic acid (%)	Tetracycline (%)	Cefazolin (%)	Imipenem (%)
*Escherichia coli*	3.1	46.2	15.9	57.6	100	76.3	73.7	67.5	45.2	2.2
*Klebsiella pneumoniae*	17.9	40	20.6	40	100	85.3	55.6	57.9	44.7	27.6
*Enterobacter aglomerance*	12	50	25	66.7	99	75	75	50	100	33.3
*Enterobacter cloacae*	0	50	0.0	50	100	50	75	55	0.0	0.0
*Klebsiella oxytoca*	0.0	100	50	50	98	50	50	0.0	0.0	0.0
*Morganellamorganii*	50	0.0	0.0	0.0	0.0	0	90	65	0.0	0.0
*Proteus vulgaris*	0.0	90	0.0	0.0	96	100	100	65	0.0	0.0
*Proteus mirabilis*	0.0	100	0.0	0.0	100	100	90	70	0.0	100

**Table 2:** The range of fosfomycin MIC in different *Enterobacteriaceae*.

Bacteria	MIC8, (*n*)	MIC16, (*n*)	MIC32, (*n*)	MIC64, (*n*)	MIC128, (*n*)	MIC256, (*n*)	MIC1024, (*n*)
*Escherichia coli,* 158 isolates	100	23	23	5	2	0	2
*Klebsiella pneumoniae,* 39 isolates	22	2	5	2	1	1	2
*Enterobacter aglomerance,* 4 isolates	3	0	1	0	0	0	0
*Enterobacter cloacae*, 2 isolates	1	1	0	0	0	0	0
*Klebsiella oxytoca*, 2 isolates	1	0	1	0	0	0	0
*Morganell amorganii*, 2 isolates	0	2	0	0	0	0	0
*Proteus vulgaris*, 2 isolates	1	0	1	0	0	0	0
*Proteus mirabilis*, 2 isolate	0	0	1	0	0	0	0

*n*, number.

**Table 3:** Different fos genes per species in this study.

Bacteria	fosC	fosX	fosA3	fosA	fosB2
*Escherichia coli*	3	2	2	0	0
*Klebsiella pneumoniae*	2	1	0	1	1
*Enterobacter aglomerance*	0	0	0	0	0
*Enterobacter cloacae*	0	0	0	0	0
*Klebsiella oxytoca*	0	0	0	0	0
*Morganell amorganii*	0	0	0	0	0
*Proteus vulgaris*	0	0	0	0	0
*Proteus mirabilis*	0	0	0	0	0

## Data Availability

The data that support the findings of this study are available within the article.
